# CsGa(HAsO_4_)_2_, the first Ga representative of the RbAl(HAsO_4_)_2_ structure type

**DOI:** 10.1107/S2056989019002081

**Published:** 2019-02-08

**Authors:** Karolina Schwendtner, Uwe Kolitsch

**Affiliations:** a Institute for Chemical Technology and Analytics, Division of Structural Chemistry, TU Wien, Getreidemarkt 9/164-SC, 1060 Vienna, Austria; b Naturhistorisches Museum, Burgring 7, 1010 Vienna, and Institut für Mineralogie und Kristallographie, Universität Wien, Althanstrasse 14, 1090 Wien, Austria

**Keywords:** crystal structure, CsGa(HAsO_4_)_2_, arsenate, hydrogen arsenate

## Abstract

The crystal structure of hydro­thermally synthesized (*T* = 493 K, 7 d) caesium gallium bis­[hydrogen arsenate(V)], CsGa(HAsO_4_)_2_, was solved by single-crystal X-ray diffraction. The compound crystallizes in the common RbAl(HAsO_4_)_2_ structure type (*R*32) and consists of a basic tetra­hedral–octa­hedral framework topology that houses Cs^+^ cations in its channels. The AsO_4_ tetra­hedron is disordered over two positions with site occupancy factors of 0.946 (1) and 0.054 (1). Strong hydrogen bonds strengthen the network. The structure was refined as inversion twin.

## Abstract 2

The tetra­hedral-octa­hedral framework crystal structure of hydro­thermally synthesized CsGa(HAsO_4_)_2_ was solved by single-crystal X-ray diffraction. CsGa(HAsO_4_)_2_ crystallizes in the polar RbAl(HAsO_4_)_2_ structure type (*R*32).

## Chemical context   

Compounds with mixed tetra­hedral–octa­hedral (T–O) framework structures are characterized by a broad range of different atomic arrangements. These topologies result in several inter­esting properties such as ion exchange (Masquelier *et al.*, 1996 ▸) and ion conductivity (Chouchene *et al.*, 2017 ▸), as well as unusual piezoelectric (Ren *et al.*, 2015 ▸), magnetic (Ouerfelli *et al.*, 2007 ▸) or non-linear optical features (frequency doubling; Sun *et al.*, 2017 ▸).

CsGa(HAsO_4_)_2_ was obtained during our extensive experimental study of the system *M* ^+^–*M* ^3+^–As^5+^–O–(H) (*M* ^+^ = Li, Na, K, Rb, Cs, Ag, Tl, NH_4_; *M* ^3+^ = Al, Ga, In, Sc, Fe, Cr, Tl), which resulted in the discovery of an unusually large variety of new structure types (Schwendtner & Kolitsch, 2004 ▸, 2005 ▸, 2007*a* ▸,*b* ▸,*c* ▸, 2017*a* ▸, 2018*a* ▸, 2019 ▸; Schwendtner, 2006 ▸). One atomic arrangement, the RbFe(HPO_4_)_2_ type (Lii & Wu, 1994 ▸; rhombohedral, *R* 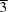 *c*), and its two relatives, the CsAl_2_As(HAsO_4_)_6_ type (Schwendtner & Kolitsch, 2018*a* ▸, rhombohedral, *R* 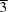 *c*) and the RbAl(HAsO_4_)_2_ type (Schwendtner & Kolitsch, 2018*a* ▸, rhombohedral, *R*32), were found to exhibit a large crystal–chemical flexibility, which allows the incorporation of a wide variety of *M* ^+^ and *M* ^3+^ cations. So far the RbFe(HPO_4_)_2_-type is represented by eight arsenate members with the following *M* ^+^ *M* ^3+^ combinations: TlAl, (NH_4_)Ga, RbIn, RbGa, RbAl, RbFe, CsIn and CsFe (Schwendtner & Kolitsch, 2017*b* ▸, 2018*a* ▸,*b* ▸,*c* ▸,*e* ▸). Six arsenates of the CsAl_2_As(HAsO_4_)_6_ type are known with the following *M* ^+^ *M* ^3+^ com­binations: RbGa, CsGa, TlGa, RbAl, CsAl and CsFe (Schwendtner & Kolitsch, 2018*a* ▸,*c* ▸,*d* ▸). CsGa(HAsO_4_)_2_ represents the third representative of the RbAl(HAsO_4_)_2_-type atomic arrangement, of which previously only the two *M* ^+^ *M* ^3+^ combinations RbAl and CsFe (Schwendtner & Kolitsch, 2018*a* ▸) were known. The 12-coordinated *M* ^+^ cations present in these types of compounds are rather large (*M =* Cs, Rb, Tl and NH_4_), with ionic radii ranging from 1.70 to 1.88 Å (Shannon, 1976 ▸). No members containing K^+^ or any smaller *M* ^+^ cations are presently known, suggesting that the ionic radius of K^+^ (1.64 Å, Shannon, 1976 ▸) is already slightly too small for this type of framework. The ionic radii of the six-coordinated *M* ^3+^ cations (*M =* Al, Cr, Fe, Ga, In) range from 0.535 to 0.800 Å (Shannon, 1976 ▸) and nearly all *M* ^3+^ cations we studied are represented in these types of compounds, with the exception of Sc^3+^ and Tl^3+^. Syntheses aimed at preparing (NH_4_)Sc(HAsO_4_)_2_, RbSc(HAsO_4_)_2_ and TlSc(HAsO_4_)_2_ instead led to the crystallization of the diarsenate compounds (NH_4_)ScAs_2_O_7_ (Kolitsch, 2004 ▸), RbScAs_2_O_7_ (Schwendtner & Kolitsch, 2004 ▸) and TlScAs_2_O_7_ (Baran *et al.*, 2006 ▸), respectively.

There exist only three other Cs–Ga arsenates: The structurally closely related CsGa_2_As(HAsO_4_)_6_ (Schwendtner & Kolitsch, 2018*b* ▸), in which one third of the *M* ^3+^O_6_ octa­hedra are replaced by AsO_6_ octa­hedra; CsGa(H_2_AsO_4_)(H_1.5_AsO_4_)_2_ (Schwendtner & Kolitsch, 2005 ▸) which was encountered in the same synthesis batch as the title compound; and Cs_2_Ga_3_(As_3_O_10_)(AsO_4_)_2_ (Lin & Lii, 1996 ▸).

## Structural commentary   

**Figure 1:**
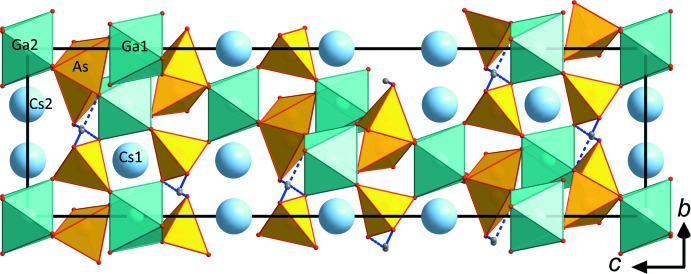
General outline of the crystal structure of CsGa(HAsO_4_)_2_ viewed along *a.* Only the main AsO_4_ tetra­hedra are shown (the As*B*-centred tetra­hedra are omitted for clarity). Hydrogen bonds are shown as blue dotted lines.

**Figure 2:**
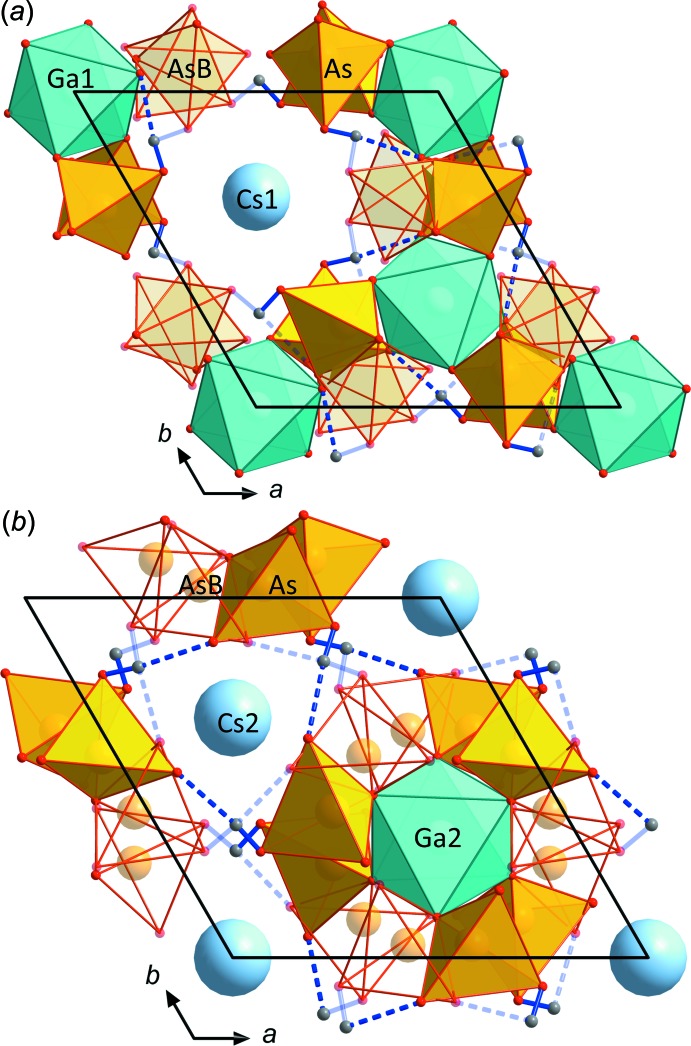
Detailed view of the different layers in the structure of CsGa(HAsO_4_)_2._ The alternative As*B*O_4_ tetra­hedra, the alternative hydrogen bonds and O*B* atoms are shown in transparent mode. (*a*) The layer showing the Ga1As_6_O_24_ group including the alternative As*B*O_4_ tetra­hedra. (*b*) The layer showing the Ga2As_6_O_24_ group including the alternative As*B*O_4_ tetra­hedra and the strongly overbonded Cs2 atom in its void.

CsGa(HAsO_4_)_2_ is a representative of the RbAl(HAsO_4_)_2_ structure type (*R*32; Schwendtner & Kolitsch, 2018*a* ▸) and has a basic tetra­hedral–octa­hedral framework structure featuring inter­penetrating channels, which host the *M* ^+^ cations (Fig. 1 ▸). This structure type is closely related to the RbFe(HPO_4_)_2_ structure type (*R* 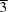 *c*; Lii & Wu, 1994 ▸), the RbAl_2_As(HAsO_4_)_6_ type (*R* 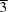 *c*; Schwendtner & Kolitsch, 2018*a* ▸) and the triclinic (NH_4_)Fe(HPO_4_)_2_ type (*P* 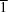; Yakubovich, 1993 ▸). The fundamental building unit in all these structure types contains *M* ^3+^O_6_ octa­hedra, which are connected *via* their six corners to six protonated AsO_4_ tetra­hedra, thereby forming an *M* ^3+^As_6_O_24_ unit. These units are in turn connected *via* three corners to other *M* ^3+^O_6_ octa­hedra. The free, protonated corner of each AsO_4_ tetra­hedron forms a medium-to-strong hydrogen bond (Table 1 ▸) to the neighbouring *M* ^3+^As_6_O_24_ group (Fig. 2 ▸ *a*,*b*). The *M* ^3+^As_6_O_24_ units are arranged in layers perpendicular to the *c*_hex_ axis (Fig. 1 ▸). The units within these layers are held together by medium–strong hydrogen bonds (Table 2 ▸). Nearly all of the representatives of these closely related structure types show pseudo-hexa­gonal to pseudo-octa­hedral crystal habits. In line with this observation, CsGa(HAsO_4_)_2_ forms tiny pseudo-hexa­gonal platelets.

The two Cs atoms in the framework voids are 12-coordi­nated. While the average Cs1—O bond length, 3.395 Å, is slightly longer than the grand mean average of 3.377 Å (Gagné & Hawthorne, 2016 ▸), it fits the low bond-valence sum (BVS) of 0.84 valence units (v.u.) which was calculated with the bond-valence parameters of Gagné & Hawthorne (2015 ▸). In contrast, the average Cs2—O bond length is slightly shorter (3.359 Å) and the individual Cs2—O bond lengths (Table 2 ▸) show a much wider bond-length range, resulting in a much too high bond-valence sum of 1.38 v.u. This is mainly caused by four very short Cs2—O bond lengths of only 3.014 Å, although even shorter Cs—O bond lengths, as low as 2.910 Å, have been reported for 12-coordinated Cs^+^ cations (Gagné & Hawthorne, 2016 ▸).

The Ga atoms at the centre of the two GaO_6_ octa­hedra are also slightly overbonded with BVSs of 3.05 and 3.07 v.u., and average Ga—O bond lengths of 1.970 and 1.967 Å for Ga1 and Ga2, respectively. These values are somewhat shorter than the grand mean average for six-coordinated Ga of 1.978 Å (Gagné & Hawthorne, 2018 ▸). The AsO_4_ tetra­hedra show the typical bond-length geometry of HAsO_4_ groups with three short and one long As—O bond. The average As—O bond length (1.689 Å) is very close to the observed average of HAsO_4_ groups (1.687 Å; Schwendtner & Kolitsch, 2019 ▸), but the As—O bond length to the protonated O4 atom (1.740 Å, Table 2 ▸) is notably longer than the average of 1.728 Å for As—OH bonds in singly protonated AsO_4_ groups (Schwendtner & Kolitsch, 2019 ▸). The BVS for the As atom is close to ideal with 4.98 v.u. All its O ligands are underbonded to a varying degree, with BVSs ranging from 1.39 v.u. for O4 to 1.92 v.u. for O1.

The As atom is characterized by a split position. The As*B* site, 1.27 Å away from the main As position, has a refined occupancy of about 5%. The As*B* site shares one apical ligand (O1) with the main AsO_4_ tetra­hedron and has three additional low-occupancy O atoms (O2*B*, O3*B* and O4*B*) as remaining ligands. The split position can roughly be explained by a mirror plane in (110). The average As*B*—O bond length of 1.684 Å is slightly shorter than the corresponding value of the main AsO_4_ tetra­hedron (1.689 Å), and the As*B*—O bonds also show a wider bond-length range (Table 2 ▸). The calculated BVS for the As*B* site (5.09 v.u.) is reasonable considering the high estimated uncertainty of this value in view of the relatively large positional and bond-length errors for the As*B* site (Table 2 ▸).

## Synthesis and crystallization   

Small pseudo-hexa­gonal colorless platelets of CsGa(HAsO_4_)_2_ were prepared hydro­thermally (*T* = 493 K, 7 d) in a Teflon-lined stainless steel autoclave from a mixture of Cs_2_CO_3_, Ga_2_O_3_ (approximate molar ratio Cs:Ga of 1:1), arsenic acid and distilled water. Enough arsenic acid was added to keep the pH between about 1.5 and 0.5. The Teflon cylinders were filled with distilled water up to approximately 80% of their inner volume. Initial and final pH values were about 1.5 and 1, respectively. The platelets were accompanied by large colourless glassy prisms of CsGa(H_2_AsO_4_)(H_1.5_AsO_4_)_2_ (Schwendtner & Kolitsch, 2005 ▸), which made up about 80% of the reaction products.

## Refinement   

Crystal data, data collection and structure refinement details are summarized in Table 3 ▸.

The refinement of CsGa(HAsO_4_)_2_ revealed a considerable residual electron-density peak of 5.1 e Å^−3^ 1.27 Å away from As and 1.62 Å away from the O1 site. The corresponding position can be generated by a mirror plane in (110) and therefore was assumed to be an alternative flipped As position (sharing the same O1 atom), similar to what was encountered in related TlAl(HAsO_4_) and CsIn(HAsO_4_)_2_ (*R* 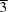 *c* type; Schwendtner & Kolitsch, 2017*b* ▸, 2018*e* ▸). An inclusion of the alternative position led to a considerable drop in the conventional *R* factor and weight parameters and the highest residual electron densities also decreased considerably. Three electron-density peaks between 1.15 and 1.19 e Å^−3^ close to this As*B* position could be attributed to the O ligands of this flipped AsO_4_ tetra­hedra and, after including them into the structure model, the conventional *R* factor dropped from 3.5 to 1.99%. The remaining highest residual electron densities of 0.72 and −0.74 e Å^−3^ are located close to the Cs positions. The occupancy of the alternative As position (Fig. 2 ▸) refined to about 5%, while the independently refined occupancy of the main As position was about 95%. For the final refinement, the displacement parameters of the As*B*, O2*B*, O3*B* and O4*B* sites were restrained to be the same as that of the main AsO_4_ tetra­hedron position, and the occupancy sums of both tetra­hedra were restrained to give a total occupancy of 1.00. The structure was refined as inversion twin with a Flack parameter of 0.46 (2).

## Supplementary Material

Crystal structure: contains datablock(s) I. DOI: 10.1107/S2056989019002081/vn2142sup1.cif

Structure factors: contains datablock(s) I. DOI: 10.1107/S2056989019002081/vn2142Isup2.hkl

CCDC reference: 1895785

Additional supporting information:  crystallographic information; 3D view; checkCIF report

## Figures and Tables

**Table 1:** Hydrogen-bond geometry (Å, °)

*D*—H⋯*A*	*D*—H	H⋯*A*	*D*⋯*A*	*D*—H⋯*A*
O4—H⋯O3^iii^	0.81 (4)	1.78 (4)	2.589 (5)	175 (6)

Symmetry code: (iii) 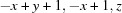.

**Table 2:** Selected bond lengths (Å)

Cs1—O4 (6×)	3.338 (3)	As—O1	1.659 (3)
Cs1—O2 (6×)	3.451 (3)	As—O2	1.667 (3)
Cs2—O4 (3×)	3.014 (3)	As—O3	1.691 (3)
Cs2—O1 (3×)	3.445 (3)	As—O4	1.740 (3)
Cs2—O4 (3×)	3.459 (3)	As*B*—O1	1.625 (7)
Cs2—O3 (3×)	3.516 (3)	As*B*—O3*B*	1.66 (6)
Ga1—O2 (3×)	1.958 (3)	As*B*—O4*B* ^i^	1.69 (6)
Ga1—O3 (3×)	1.982 (3)	As*B*—O2*B* ^ii^	1.76 (7)
Ga2—O1 (6×)	1.967 (3)		

Symmetry codes: (i) 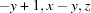; (ii) 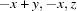.

**Table 3:** Experimental details

Crystal data	
Chemical formula	CsGa(HAsO_4_)_2_
*M*_r_	482.49
Crystal system, space group	Trigonal, *R*32:*H*
Temperature (K)	293
*a*, *c* (Å)	8.481 (1), 27.050 (5)
*V* (Å^3^)	1685.0 (5)
*Z*	9
Radiation type	Mo *K*α
μ (mm^−1^)	17.24
Crystal size (mm)	0.03 × 0.03 × 0.01

Data collection	
Diffractometer	Nonius KappaCCD single-crystal four-circle
Absorption correction	Multi-scan (*HKL* *SCALEPACK*; Otwinowski *et al.*, 2003 ▸)
*T*_min_, *T*_max_	0.626, 0.846
No. of measured, independent and observed [*I* > 2σ(*I*)] reflections	2738, 1375, 1283
*R*_int_	0.018
(sin θ/λ)_max_ (Å^−1^)	0.757

Refinement	
*R*[*F* ^2^ > 2σ(*F* ^2^)], *wR*(*F* ^2^), *S*	0.019, 0.042, 1.07
No. of reflections	1375
No. of parameters	76
No. of restraints	2
H-atom treatment	All H-atom parameters refined
Δρ_max_, Δρ_min_ (e Å^−3^)	0.72, −0.74
Absolute structure	Refined as an inversion twin
Absolute structure parameter	0.46 (2)

Computer programs: *COLLECT* (Nonius, 2003 ▸), *HKL* *DENZO* and *SCALEPACK* (Otwinowski *et al.*, 2003 ▸), *SHELXS97* (Sheldrick, 2008 ▸), *SHELXL2016* (Sheldrick, 2015 ▸), *DIAMOND* (Brandenburg, 2005 ▸) and *publCIF* (Westrip, 2010 ▸).

## supplementary crystallographic information

### Crystal data

**Table:**

CsGa(HAsO_4_)_2_	*D*_x_ = 4.279 Mg m^−^^3^
*M**_r_* = 482.49	Mo *K*α radiation, λ = 0.71073 Å
Trigonal, *R*32:*H*	Cell parameters from 1370 reflections
*a* = 8.481 (1) Å	θ = 2.3–32.5°
*c* = 27.050 (5) Å	µ = 17.24 mm^−^^1^
*V* = 1685.0 (5) Å^3^	*T* = 293 K
*Z* = 9	Tiny hexagonal platelets, colourless
*F*(000) = 1962	0.03 × 0.03 × 0.01 mm

### Data collection

**Table:**

Nonius KappaCCD single-crystal four-circle diffractometer	1283 reflections with *I* > 2σ(*I*)
Radiation source: fine-focus sealed tube	*R*_int_ = 0.018
φ and ω scans	θ_max_ = 32.5°, θ_min_ = 2.3°
Absorption correction: multi-scan (HKL SCALEPACK; Otwinowski *et al.*, 2003)	*h* = −12→12
*T*_min_ = 0.626, *T*_max_ = 0.846	*k* = −10→10
2738 measured reflections	*l* = −40→40
1375 independent reflections	

### Refinement

**Table:**

Refinement on *F*^2^	Hydrogen site location: difference Fourier map
Least-squares matrix: full	All H-atom parameters refined
*R*[*F*^2^ > 2σ(*F*^2^)] = 0.019	*w* = 1/[σ^2^(*F*_o_^2^) + (0.0194*P*)^2^ + 2.6152*P*] where *P* = (*F*_o_^2^ + 2*F*_c_^2^)/3
*wR*(*F*^2^) = 0.042	(Δ/σ)_max_ < 0.001
*S* = 1.07	Δρ_max_ = 0.72 e Å^−^^3^
1375 reflections	Δρ_min_ = −0.74 e Å^−^^3^
76 parameters	Extinction correction: SHELXL2016 (Sheldrick, 2015), Fc^*^=kFc[1+0.001xFc^2^λ^3^/sin(2θ)]^-1/4^
2 restraints	Extinction coefficient: 0.00041 (4)
Primary atom site location: structure-invariant direct methods	Absolute structure: Refined as an inversion twin
Secondary atom site location: difference Fourier map	Absolute structure parameter: 0.46 (2)

### Special details

**Table:**

Geometry. All esds (except the esd in the dihedral angle between two l.s. planes) are estimated using the full covariance matrix. The cell esds are taken into account individually in the estimation of esds in distances, angles and torsion angles; correlations between esds in cell parameters are only used when they are defined by crystal symmetry. An approximate (isotropic) treatment of cell esds is used for estimating esds involving l.s. planes.
Refinement. Refined as a 2-component inversion twin.

### Fractional atomic coordinates and isotropic or equivalent isotropic displacement parameters (Å^2^)

**Table:**

	*x*	*y*	*z*	*U*_iso_*/*U*_eq_	Occ. (<1)
O1	0.2225 (4)	0.1039 (5)	0.04039 (9)	0.0161 (5)	
Cs1	0.333333	0.666667	0.166667	0.02717 (16)	
Cs2	0.333333	0.666667	0.00046 (2)	0.02105 (12)	
Ga1	0.000000	0.000000	0.17439 (2)	0.00792 (13)	
Ga2	0.000000	0.000000	0.000000	0.00791 (17)	
As	0.29653 (6)	0.22380 (6)	0.09219 (2)	0.00852 (10)	0.9461 (12)
O2	0.1465 (4)	0.2152 (5)	0.13347 (13)	0.0110 (6)	0.9461 (12)
O3	0.4566 (4)	0.1811 (5)	0.11541 (11)	0.0110 (5)	0.9461 (12)
O4	0.4135 (4)	0.4521 (4)	0.07510 (12)	0.0151 (5)	0.9461 (12)
AsB	0.2984 (10)	0.0710 (10)	0.0923 (3)	0.00852 (10)	0.0540 (12)
O2B	0.081 (9)	0.213 (10)	0.134 (2)	0.0110 (6)	0.0540 (12)
O3B	0.457 (7)	0.265 (8)	0.117 (2)	0.0110 (5)	0.0540 (12)
O4B	0.549 (8)	0.587 (7)	0.077 (2)	0.0151 (5)	0.0540 (12)
H	0.510 (7)	0.474 (8)	0.0874 (17)	0.017 (13)*	

### Atomic displacement parameters (Å^2^)

**Table:**

	*U*^11^	*U*^22^	*U*^33^	*U*^12^	*U*^13^	*U*^23^
O1	0.0123 (12)	0.0253 (16)	0.0101 (11)	0.0090 (14)	−0.0040 (9)	−0.0056 (13)
Cs1	0.0313 (2)	0.0313 (2)	0.0188 (3)	0.01567 (12)	0.000	0.000
Cs2	0.02470 (15)	0.02470 (15)	0.01374 (19)	0.01235 (8)	0.000	0.000
Ga1	0.00859 (18)	0.00859 (18)	0.0066 (3)	0.00430 (9)	0.000	0.000
Ga2	0.0088 (3)	0.0088 (3)	0.0062 (4)	0.00438 (13)	0.000	0.000
As	0.00777 (17)	0.01075 (19)	0.00680 (16)	0.00446 (15)	−0.00031 (14)	0.00020 (14)
O2	0.0107 (14)	0.0127 (13)	0.0097 (12)	0.0060 (13)	0.0045 (11)	0.0008 (10)
O3	0.0110 (13)	0.0138 (14)	0.0104 (12)	0.0078 (10)	−0.0029 (11)	−0.0029 (11)
O4	0.0103 (14)	0.0123 (14)	0.0207 (14)	0.0042 (12)	−0.0013 (12)	0.0052 (12)
AsB	0.00777 (17)	0.01075 (19)	0.00680 (16)	0.00446 (15)	−0.00031 (14)	0.00020 (14)
O2B	0.0107 (14)	0.0127 (13)	0.0097 (12)	0.0060 (13)	0.0045 (11)	0.0008 (10)
O3B	0.0110 (13)	0.0138 (14)	0.0104 (12)	0.0078 (10)	−0.0029 (11)	−0.0029 (11)
O4B	0.0103 (14)	0.0123 (14)	0.0207 (14)	0.0042 (12)	−0.0013 (12)	0.0052 (12)

### Geometric parameters (Å, º)

**Table:**

O1—AsB	1.625 (7)	Ga1—O3^iv^	1.982 (3)
O1—As	1.659 (3)	Ga1—O3^xi^	1.982 (3)
O1—Ga2	1.967 (3)	Ga1—O3^xii^	1.982 (3)
O1—Cs2^i^	3.445 (3)	As—O2	1.667 (3)
Cs1—O4	3.338 (3)	As—O3	1.691 (3)
Cs1—O4^ii^	3.338 (3)	As—O4	1.740 (3)
Cs1—O4^iii^	3.338 (3)	As—H	1.99 (6)
Cs1—O4^iv^	3.338 (3)	O4—H	0.81 (4)
Cs1—O4^v^	3.338 (3)	AsB—O3B	1.66 (6)
Cs1—O4^vi^	3.338 (3)	AsB—O4B^xiii^	1.69 (6)
Cs1—O2^iv^	3.451 (3)	AsB—O2B^x^	1.76 (7)
Cs1—O2^ii^	3.451 (3)	O4B—H	0.88 (7)
Cs1—O2	3.451 (3)	Cs1—O4 (6x)	3.338 (3)
Cs1—O2^iii^	3.451 (3)	Cs1—O2 (6x)	3.451 (3)
Cs1—O2^v^	3.451 (3)	Cs2—O4 (3x)	3.014 (3)
Cs1—O2^vi^	3.451 (3)	Cs2—O1 (3x)	3.445 (3)
Cs1—H	3.46 (5)	Cs2—O4 (3x)	3.459 (3)
Cs2—O4^iii^	3.014 (3)	Cs2—O3 (3x)	3.516 (3)
Cs2—O4^ii^	3.014 (3)	Ga1—O2 (3x)	1.958 (3)
Cs2—O4	3.014 (3)	Ga1—O3 (3x)	1.982 (3)
Cs2—O4^vii^	3.459 (3)	Ga2—O1 (6x)	1.967 (3)
Cs2—O4^viii^	3.459 (3)	As—O1	1.659 (3)
Cs2—O4^i^	3.459 (3)	As—O2	1.667 (3)
Cs2—O3^i^	3.516 (3)	As—O3	1.691 (3)
Cs2—O3^vii^	3.516 (3)	As—O4	1.740 (3)
Cs2—O3^viii^	3.516 (3)	AsB—O1	1.625 (7)
Ga1—O2^ix^	1.958 (3)	AsB—O3B	1.66 (6)
Ga1—O2	1.958 (3)	AsB—O4B^xiii^	1.69 (6)
Ga1—O2^x^	1.958 (3)	AsB—O2B^x^	1.76 (7)
			
As—O1—Ga2	136.69 (19)	O1^vii^—Cs2—O4^i^	124.15 (7)
AsB—O1—Cs2^i^	87.7 (3)	O1^i^—Cs2—O4^i^	46.56 (8)
As—O1—Cs2^i^	87.31 (11)	O1^viii^—Cs2—O4^i^	63.74 (8)
Ga2—O1—Cs2^i^	127.46 (10)	O4^vii^—Cs2—O4^i^	88.64 (8)
O4—Cs1—O4^ii^	70.99 (9)	O4^viii^—Cs2—O4^i^	88.64 (8)
O4—Cs1—O4^iii^	70.99 (9)	O4^iii^—Cs2—O3^i^	159.00 (8)
O4^ii^—Cs1—O4^iii^	70.99 (9)	O4^ii^—Cs2—O3^i^	115.45 (8)
O4—Cs1—O4^iv^	99.40 (11)	O4—Cs2—O3^i^	115.32 (8)
O4^ii^—Cs1—O4^iv^	123.13 (11)	O1^vii^—Cs2—O3^i^	80.58 (7)
O4^iii^—Cs1—O4^iv^	160.35 (10)	O1^i^—Cs2—O3^i^	45.29 (7)
O4—Cs1—O4^v^	123.13 (11)	O1^viii^—Cs2—O3^i^	90.53 (7)
O4^ii^—Cs1—O4^v^	160.35 (10)	O4^vii^—Cs2—O3^i^	43.57 (8)
O4^iii^—Cs1—O4^v^	99.40 (11)	O4^viii^—Cs2—O3^i^	80.71 (8)
O4^iv^—Cs1—O4^v^	70.99 (9)	O4^i^—Cs2—O3^i^	46.05 (8)
O4—Cs1—O4^vi^	160.35 (10)	O4^iii^—Cs2—O3^vii^	115.45 (8)
O4^ii^—Cs1—O4^vi^	99.40 (11)	O4^ii^—Cs2—O3^vii^	115.32 (8)
O4^iii^—Cs1—O4^vi^	123.13 (11)	O4—Cs2—O3^vii^	159.00 (8)
O4^iv^—Cs1—O4^vi^	70.99 (9)	O1^vii^—Cs2—O3^vii^	45.29 (7)
O4^v^—Cs1—O4^vi^	70.99 (9)	O1^i^—Cs2—O3^vii^	90.53 (7)
O4—Cs1—O2^iv^	63.50 (8)	O1^viii^—Cs2—O3^vii^	80.58 (7)
O4^ii^—Cs1—O2^iv^	126.94 (7)	O4^vii^—Cs2—O3^vii^	46.05 (8)
O4^iii^—Cs1—O2^iv^	115.11 (8)	O4^viii^—Cs2—O3^vii^	43.57 (8)
O4^iv^—Cs1—O2^iv^	46.21 (8)	O4^i^—Cs2—O3^vii^	80.71 (8)
O4^v^—Cs1—O2^iv^	72.50 (8)	O3^i^—Cs2—O3^vii^	46.22 (9)
O4^vi^—Cs1—O2^iv^	114.43 (8)	O4^iii^—Cs2—O3^viii^	115.32 (8)
O4—Cs1—O2^ii^	114.43 (8)	O4^ii^—Cs2—O3^viii^	159.00 (8)
O4^ii^—Cs1—O2^ii^	46.21 (8)	O4—Cs2—O3^viii^	115.45 (8)
O4^iii^—Cs1—O2^ii^	72.51 (8)	O1^vii^—Cs2—O3^viii^	90.53 (7)
O4^iv^—Cs1—O2^ii^	126.94 (8)	O1^i^—Cs2—O3^viii^	80.58 (7)
O4^v^—Cs1—O2^ii^	115.11 (8)	O1^viii^—Cs2—O3^viii^	45.29 (7)
O4^vi^—Cs1—O2^ii^	63.50 (8)	O4^vii^—Cs2—O3^viii^	80.71 (8)
O2^iv^—Cs1—O2^ii^	169.02 (11)	O4^viii^—Cs2—O3^viii^	46.05 (8)
O4—Cs1—O2	46.21 (8)	O4^i^—Cs2—O3^viii^	43.57 (8)
O4^ii^—Cs1—O2	72.50 (8)	O3^i^—Cs2—O3^viii^	46.22 (9)
O4^iii^—Cs1—O2	114.43 (8)	O3^vii^—Cs2—O3^viii^	46.22 (9)
O4^iv^—Cs1—O2	63.50 (8)	O2^ix^—Ga1—O2	91.18 (14)
O4^v^—Cs1—O2	126.94 (7)	O2^ix^—Ga1—O2^x^	91.18 (14)
O4^vi^—Cs1—O2	115.11 (8)	O2—Ga1—O2^x^	91.18 (14)
O2^iv^—Cs1—O2	56.73 (11)	O2^ix^—Ga1—O3^iv^	176.98 (13)
O2^ii^—Cs1—O2	113.48 (5)	O2—Ga1—O3^iv^	91.84 (14)
O4—Cs1—O2^iii^	72.50 (8)	O2^x^—Ga1—O3^iv^	88.69 (14)
O4^ii^—Cs1—O2^iii^	114.43 (8)	O2^ix^—Ga1—O3^xi^	88.69 (14)
O4^iii^—Cs1—O2^iii^	46.21 (8)	O2—Ga1—O3^xi^	176.98 (13)
O4^iv^—Cs1—O2^iii^	115.11 (8)	O2^x^—Ga1—O3^xi^	91.84 (14)
O4^v^—Cs1—O2^iii^	63.50 (9)	O3^iv^—Ga1—O3^xi^	88.30 (14)
O4^vi^—Cs1—O2^iii^	126.94 (8)	O2^ix^—Ga1—O3^xii^	91.84 (14)
O2^iv^—Cs1—O2^iii^	76.70 (11)	O2—Ga1—O3^xii^	88.69 (14)
O2^ii^—Cs1—O2^iii^	113.48 (5)	O2^x^—Ga1—O3^xii^	176.98 (13)
O2—Cs1—O2^iii^	113.48 (5)	O3^iv^—Ga1—O3^xii^	88.30 (14)
O4—Cs1—O2^v^	126.94 (8)	O3^xi^—Ga1—O3^xii^	88.30 (14)
O4^ii^—Cs1—O2^v^	115.11 (8)	O2^ix^—Ga1—Cs2^xiv^	124.43 (10)
O4^iii^—Cs1—O2^v^	63.50 (9)	O2—Ga1—Cs2^xiv^	124.43 (10)
O4^iv^—Cs1—O2^v^	114.43 (8)	O2^x^—Ga1—Cs2^xiv^	124.43 (10)
O4^v^—Cs1—O2^v^	46.21 (8)	O3^iv^—Ga1—Cs2^xiv^	53.54 (10)
O4^vi^—Cs1—O2^v^	72.50 (8)	O3^xi^—Ga1—Cs2^xiv^	53.54 (10)
O2^iv^—Cs1—O2^v^	113.48 (5)	O3^xii^—Ga1—Cs2^xiv^	53.54 (10)
O2^ii^—Cs1—O2^v^	76.70 (11)	O1—Ga2—O1^xv^	176.3 (2)
O2—Cs1—O2^v^	169.02 (11)	O1—Ga2—O1^ix^	92.14 (11)
O2^iii^—Cs1—O2^v^	56.74 (11)	O1^xv^—Ga2—O1^ix^	90.55 (19)
O4—Cs1—O2^vi^	115.11 (8)	O1—Ga2—O1^xvi^	85.29 (19)
O4^ii^—Cs1—O2^vi^	63.50 (8)	O1^xv^—Ga2—O1^xvi^	92.13 (11)
O4^iii^—Cs1—O2^vi^	126.94 (7)	O1^ix^—Ga2—O1^xvi^	176.3 (2)
O4^iv^—Cs1—O2^vi^	72.50 (8)	O1—Ga2—O1^x^	92.14 (11)
O4^v^—Cs1—O2^vi^	114.43 (8)	O1^xv^—Ga2—O1^x^	85.29 (19)
O4^vi^—Cs1—O2^vi^	46.21 (8)	O1^ix^—Ga2—O1^x^	92.13 (11)
O2^iv^—Cs1—O2^vi^	113.48 (5)	O1^xvi^—Ga2—O1^x^	90.55 (19)
O2^ii^—Cs1—O2^vi^	56.73 (11)	O1—Ga2—O1^i^	90.55 (19)
O2—Cs1—O2^vi^	76.70 (11)	O1^xv^—Ga2—O1^i^	92.13 (11)
O2^iii^—Cs1—O2^vi^	169.02 (11)	O1^ix^—Ga2—O1^i^	85.29 (19)
O2^v^—Cs1—O2^vi^	113.48 (5)	O1^xvi^—Ga2—O1^i^	92.13 (11)
O4—Cs1—H	13.6 (7)	O1^x^—Ga2—O1^i^	176.3 (2)
O4^ii^—Cs1—H	84.3 (7)	O1—As—O2	119.51 (15)
O4^iii^—Cs1—H	71.9 (10)	O1—As—O3	106.29 (15)
O4^iv^—Cs1—H	94.7 (9)	O2—As—O3	114.75 (17)
O4^v^—Cs1—H	109.6 (7)	O1—As—O4	106.75 (16)
O4^vi^—Cs1—H	164.9 (10)	O2—As—O4	102.97 (16)
O2^iv^—Cs1—H	53.6 (8)	O3—As—O4	105.36 (17)
O2^ii^—Cs1—H	126.1 (7)	O1—As—Cs2^i^	66.48 (10)
O2—Cs1—H	51.8 (10)	O2—As—Cs2^i^	169.73 (12)
O2^iii^—Cs1—H	62.9 (9)	O3—As—Cs2^i^	68.86 (11)
O2^v^—Cs1—H	119.3 (10)	O4—As—Cs2^i^	66.81 (11)
O2^vi^—Cs1—H	126.0 (9)	O1—As—Cs1	143.32 (12)
O4^iii^—Cs2—O4^ii^	80.04 (10)	O2—As—Cs1	54.72 (12)
O4^iii^—Cs2—O4	80.04 (10)	O3—As—Cs1	108.02 (11)
O4^ii^—Cs2—O4	80.04 (10)	O4—As—Cs1	51.41 (11)
O4^iii^—Cs2—O1^vii^	90.97 (8)	Cs2^i^—As—Cs1	115.260 (12)
O4^ii^—Cs2—O1^vii^	74.30 (8)	O1—As—H	117.5 (13)
O4—Cs2—O1^vii^	153.94 (8)	O2—As—H	110.9 (13)
O4^iii^—Cs2—O1^i^	153.94 (8)	O3—As—H	81.6 (12)
O4^ii^—Cs2—O1^i^	90.97 (8)	O4—As—H	24.0 (12)
O4—Cs2—O1^i^	74.30 (8)	Cs2^i^—As—H	59.4 (13)
O1^vii^—Cs2—O1^i^	110.22 (4)	Cs1—As—H	56.4 (13)
O4^iii^—Cs2—O1^viii^	74.30 (8)	As—O2—Ga1	122.30 (19)
O4^ii^—Cs2—O1^viii^	153.94 (8)	As—O2—Cs1	102.06 (14)
O4—Cs2—O1^viii^	90.97 (8)	Ga1—O2—Cs1	127.77 (14)
O1^vii^—Cs2—O1^viii^	110.22 (4)	As—O3—Ga1^xvii^	129.62 (19)
O1^i^—Cs2—O1^viii^	110.22 (4)	As—O3—Cs2^i^	84.49 (12)
O4^iii^—Cs2—O4^vii^	136.20 (4)	Ga1^xvii^—O3—Cs2^i^	99.51 (12)
O4^ii^—Cs2—O4^vii^	78.31 (9)	As—O4—Cs2	132.43 (15)
O4—Cs2—O4^vii^	131.91 (5)	As—O4—Cs1	104.56 (13)
O1^vii^—Cs2—O4^vii^	46.56 (8)	Cs2—O4—Cs1	89.95 (9)
O1^i^—Cs2—O4^vii^	63.74 (8)	As—O4—Cs2^i^	85.66 (12)
O1^viii^—Cs2—O4^vii^	124.15 (7)	Cs2—O4—Cs2^i^	98.06 (9)
O4^iii^—Cs2—O4^viii^	78.31 (9)	Cs1—O4—Cs2^i^	157.27 (10)
O4^ii^—Cs2—O4^viii^	131.91 (5)	As—O4—H	96 (4)
O4—Cs2—O4^viii^	136.20 (4)	Cs2—O4—H	130 (4)
O1^vii^—Cs2—O4^viii^	63.74 (8)	Cs1—O4—H	92 (3)
O1^i^—Cs2—O4^viii^	124.15 (7)	Cs2^i^—O4—H	67 (3)
O1^viii^—Cs2—O4^viii^	46.56 (8)	O1—AsB—O3B	112 (2)
O4^vii^—Cs2—O4^viii^	88.64 (8)	O1—AsB—O4B^xiii^	105.6 (18)
O4^iii^—Cs2—O4^i^	131.91 (5)	O3B—AsB—O4B^xiii^	104 (3)
O4^ii^—Cs2—O4^i^	136.20 (4)	O1—AsB—O2B^x^	116 (2)
O4—Cs2—O4^i^	78.32 (9)	AsB^xviii^—O4B—H	105 (6)

Symmetry codes: (i) *y*, *x*, −*z*; (ii) −*x*+*y*, −*x*+1, *z*; (iii) −*y*+1, *x*−*y*+1, *z*; (iv) −*x*+2/3, −*x*+*y*+1/3, −*z*+1/3; (v) *x*−*y*+2/3, −*y*+4/3, −*z*+1/3; (vi) *y*−1/3, *x*+1/3, −*z*+1/3; (vii) *x*−*y*, −*y*+1, −*z*; (viii) −*x*+1, −*x*+*y*+1, −*z*; (ix) −*y*, *x*−*y*, *z*; (x) −*x*+*y*, −*x*, *z*; (xi) *y*−1/3, *x*−2/3, −*z*+1/3; (xii) *x*−*y*−1/3, −*y*+1/3, −*z*+1/3; (xiii) −*y*+1, *x*−*y*, *z*; (xiv) *x*−1/3, *y*−2/3, *z*+1/3; (xv) −*x*, −*x*+*y*, −*z*; (xvi) *x*−*y*, −*y*, −*z*; (xvii) *y*+2/3, *x*+1/3, −*z*+1/3; (xviii) −*x*+*y*+1, −*x*+1, *z*.

### Hydrogen-bond geometry (Å, º)

**Table:**

*D*—H···*A*	*D*—H	H···*A*	*D*···*A*	*D*—H···*A*
O4—H···O3^xviii^	0.81 (4)	1.78 (4)	2.589 (5)	175 (6)

Symmetry code: (xviii) −*x*+*y*+1, −*x*+1, *z*.
